# Fluconazole Population Pharmacokinetics after Fosfluconazole Administration and Dosing Optimization in Extremely Low-Birth-Weight Infants

**DOI:** 10.1128/spectrum.01952-21

**Published:** 2022-03-10

**Authors:** Ayano Tanzawa, Jumpei Saito, Kensuke Shoji, Yuka Kojo, Takanori Funaki, Hidehiko Maruyama, Tetsuya Isayama, Yushi Ito, Hidefumi Nakamura, Akimasa Yamatani

**Affiliations:** a Department of Pharmacy, National Center for Child Health and Development, Tokyo, Japan; b Division of Infectious Diseases, Department of Medical Subspecialties, National Center for Child Health and Development, Tokyo, Japan; c Division of Neonatology, Center for Maternal-Fetal, Neonatal and Reproductive Medicine, National Center for Child Health and Development, Tokyo, Japan; d Department of Research and Development Supervision, National Center for Child Health and Development, Tokyo, Japan; University of Lagos

**Keywords:** fosfluconazole, fluconazole, extremely low birth weight, infants

## Abstract

A prospective single-center study was conducted to characterize the pharmacokinetics (PK) of fluconazole (FLCZ) in extremely low-birth-weight infants (ELBWIs) who received fosfluconazole (F-FLCZ). Intravenous F-FLCZ was administered at a dose of 3 mg/kg of body weight every 72 h during the first 2 weeks of life, every 48 h during the third and fourth weeks of life, and every 24 h after 5 weeks of life. Blood samples from ELBWIs treated with F-FLCZ were collected using scavenged samples. The concentration of FLCZ was determined using liquid chromatography-tandem mass spectrometry. The population pharmacokinetic model was established using Phenix NLME 8.2 software. In total, 18 ELBWIs were included in this analysis. Individual PK parameters were determined by a one-compartment analysis with first-order conversion. Postmenstrual age (PMA), serum creatinine (SCr), and alkaline phosphatase were considered covariates for clearance (CL). The mean population CL and the volume of distribution were 0.011 L/h/kg^0.75^ and 0.95 L/kg, respectively. Simulation assessments with the final model revealed that the current regimen (3 mg/kg every 72 h) could achieve the proposed target FLCZ trough concentration (>2 μg/mL) in 43.3% and 72.2% of infants with a PMA of ≥37 and 30 to 36 weeks, respectively, and an SCr level of <0.5 mg/dL. Shortened dosing intervals (every 48 or 24 h) might improve the probability of target attainment. This study was the first to assess the PK of F-FLCZ in ELBWI, as well as the first to provide fundamental information about FLCZ exposure after F-FLCZ administration, with the goal of facilitating dose optimization in the ELBWI population.

**IMPORTANCE** Invasive fungal infection is an important cause of mortality and morbidity in very preterm or very-low-birth-weight infants. In order to limit the risk of invasive fungal infections in this population, the administration of fluconazole is generally recommended for extremely low-birth-weight infants admitted to a neonatal intensive care unit with a Candida species colonization prevalence rate of >10%, under the guidelines of the Infectious Diseases Society of America. Fosfluconazole can reduce the volume of solution required for intravenous therapy compared to fluconazole because it has increased solubility, which is a major advantage for infants undergoing strict fluid management. To date, no study has demonstrated the fluconazole pharmacokinetics after fosfluconazole administration in neonates and infants, and this needs to be clarified. Here, we characterized the pharmacokinetics of fluconazole in extremely low-birth-weight infants who received F-FLCZ and explored the appropriate dosage in this patient population.

## INTRODUCTION

The incidence of invasive fungal infection ranges from 2% to 16% among infants with very low birth weight (VLBW; <1,500 g) (1, 2), and it is higher in infants with lower birth weight and/or prematurity (3). The mortality rate of invasive Candida infections in extremely low-birth-weight infants (ELBWIs; <1,000 g) ranges from 30% to 40%, and neurodevelopmental impairment is common among survivors (4, 5). Although the universal use of antifungal prophylaxis remains controversial, the administration of fluconazole (FLCZ)—an antifungal prophylaxis—is generally recommended for ELBWIs admitted to a neonatal intensive care unit (NICU) with a Candida species colonization prevalence rate of >10%, according to the guidelines of the Infectious Diseases Society of America (6).

Fosfluconazole (F-FLCZ) is a phosphate ester prodrug of FLCZ that can increase the solubility and reduce the volume of solutions required for intravenous therapy. It is rapidly converted to FLCZ by alkaline phosphatase (ALP) in the tissues and blood among adult patients (7). ALP appears to be abundant in infants (8), and fosphenytoin—a phosphate-ester prodrug of phenytoin—is rapidly converted to phenytoin (9). Therefore, it is conceivable that F-FLCZ is also rapidly converted to FLCZ for this population. F-FLCZ is increasingly applied as an antifungal prophylaxis among ELBWIs in routine clinical care at several institutions across Japan, including at our center (10, 11). Given that the acceptable volume of infused fluid is smaller, this treatment option appears beneficial for infants on strict fluid management, particularly among low-birth-weight or preterm infants. The current dosage of F-FLCZ is estimated from the pharmacokinetics (PK) of FLCZ after intravenous F-FLCZ administration in adults (12, 13) and FLCZ PK after FLCZ administration in infants (14, 15). To date, no study has demonstrated the FLCZ PK after F-FLCZ administration in infants. The primary aim of the present study was to characterize FLCZ PK in ELBWIs who received F-FLCZ, as well as to explore the appropriate dosage in this patient population. We have suggested the appropriate dosage for this group population based on the results of our analysis.

## RESULTS

### Clinical characteristics of participants.

Eighteen ELBWIs who were admitted to the NICU and received F-FLCZ were enrolled in the current study. The demographic and clinical characteristics of the enrolled patients are depicted in Table 1. During prophylactic treatment with F-FLCZ, amikacin, indomethacin, and vancomycin were found to be the major concomitant drugs that could affect the FLCZ PK, with renal function as the main elimination pathway. Of 18 patients, 17 (94.4%) were treated with amikacin for possible infection, 8 (44.4%) with indomethacin for patent ductus arteriosus, and 3 (16.7%) with vancomycin for catheter-related bloodstream infection. The median duration of F-FLCZ for prophylactic treatment was 17 days (interquartile range [IQR], 11 to 21 days), and none of the infants developed Candida infections. The aspartate aminotransferase (AST) and alanine aminotransferase (ALT) levels at the baseline were 46 (IQR, 32 to 74) and 3 (IQR, 3 to 6) IU/L, respectively. For the entire study duration, the median AST and ALT levels were 37 (IQR, 26 to 69) and 7 (IQR, 3 to 13) IU/L, respectively. The AST levels of the two patients with severe neonatal asphyxia were >250 IU/L during the study period, and there was no significant adverse event related to FLCZ. F-FLCZ administration was not discontinued regardless of the possible adverse effects in all patients.

**TABLE 1:** Demographic and clinical characteristics of the patients

Characteristic^*a*^	No. (%) or median (IQR) (*n* = 18)^*b*^
Male sex	11 (61.1)
PNA (days)	0 (0–0)^*c*^
	5.4 (6–35)^*d*^
GA (wks)	23.2 (23.1–26.4)
PMA (wks)	28.5 (26.3–31.7)^*d*^
BW (g)	748 (521–866)
WT (g)	750 (580–923)^*d*^
HT (cm)	31.0 (28.0–32.5)^*d*^
SCr (mg/dL)	0.60 (0.51–0.66)^*c*^
	0.64 (0.48–1.07)^*d*^
SCr of mothers (mg/dL)	0.48 (0.47–0.51)
Alb (g/dL)	2.7 (2.4–2.6)^*d*^
ALP (IU/mL)	958 (672–1,208)^*d*^
AST (IU/mL)	46 (32–74)^*c*^
	37 (26–69)^*d*^
ALT (IU/mL)	3 (3–6)^*c*^
	7 (4–13)^*d*^
Duration of fosfluconazole treatment (days)	17 (11–21)
Scavenged serum samples used to analyze fluconazole concn	442 (100)
Sample collection time after dose (h)	42.2 (18.5–61.5)

a PNA, postnatal age; GA, gestational age; PMA, postmenstrual age; BW, birth weight; WT, current body weight; HT, current height; SCr, serum creatinine; Alb, albumin; ALP, alkaline phosphatase; AST, aspartate aminotransferase; ALT, alanine aminotransferase.

b IQR, interquartile range.

c On the fosfluconazole starting day.

d Entire study period.

### PK specimens.

**FIG 1:**
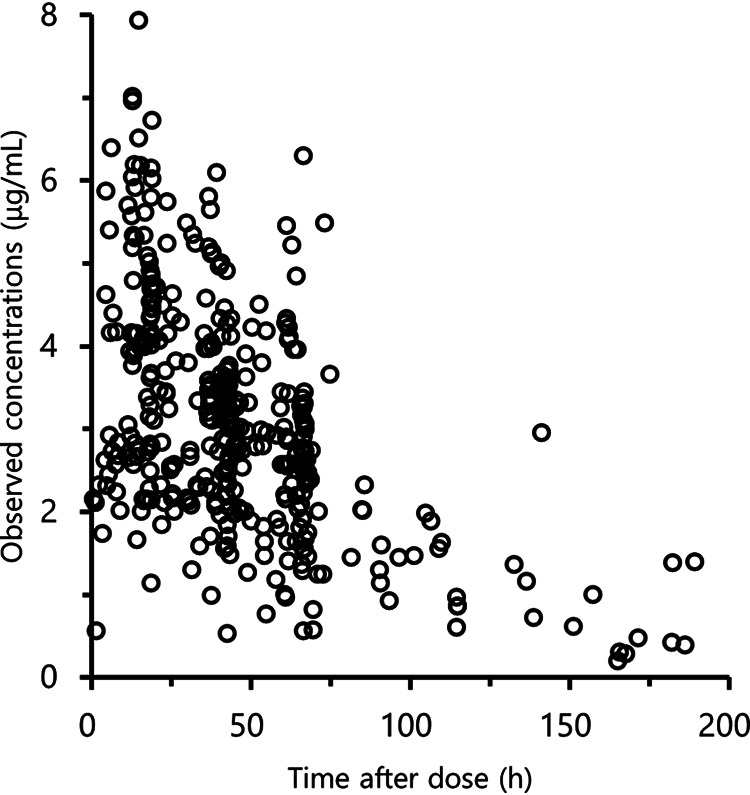
Fluconazole concentration-time profile after fosfluconazole administration.

In total, 587 scavenged samples, with a median of 17 samples per patient (IQR, 12 to 26 samples), were collected for 18 infants. Among these, 442 samples were subjected to drug concentration measurement because 145 of them did not have the required serum sample volume for measurement. Figure 1 shows the FLCZ concentrations used in the PK analysis. Data after the first dose were available for all patients, with a total of 58 data points (mean number of samples per patient, 3.6 [range, 1 to 6]). The median time to PK sampling was 42.2 h (IQR, 18.5 to 61.5 h) after the latest administration. The median concentration was 2.9 μg/mL (IQR, 2.2 to 4.1 μg/mL). In total, 64 (14.4%) samples were excluded from further analysis because they were found to be below the limit of quantitation (<0.0031 μg/mL).

### Population PK (PPK) model building.

The preliminary analysis exploring the base structure model revealed that the objective function values (OFVs) in the one- and two-compartment models were 819.6 and 818.8, respectively. Hence, for further analyses, the one-compartment model was used. The model combining proportional and additive errors best described the residual variability. Meanwhile, the exponential error model was selected to assume interindividual variability. The interindividual random effect on the conversion rate constant of F-FLCZ to FLCZ (*k_c_*) was excluded in the model building process because the shrinkage factor of *k_c_* was 0.9. Thus, the minor interindividual variability of parameters could be eliminated without significantly altering the OFV. The η shrinkage for clearance (CL) and the volume of distribution (*V*) in the base model was low (<0.3), which suggested that the estimates were not overparameterized. In the univariate analysis, after the inclusion of allometric body weight (WT) as a default for CL and *V*, the inclusion of postmenstrual age (PMA), postnatal age (PNA), serum creatinine (SCr), height (HT), and ALP as covariates of CL resulted in significant reductions in OFV, by 310.2, 234.3, 140.8, 162.9, and 102.7, respectively. Given that PMA and PNA were strongly correlated, PNA was excluded from the covariates for CL. For *V*, the inclusion of SCr, albumin (Alb), and ALP led to significant reductions in OFV, by 21.8, 12.8, and 13.5, respectively. However, the residual standard errors for the selected covariates were found to be 54.2%, 66.6%, and 57.2%, respectively. Furthermore, the null value was included in the estimated 95% confidence intervals (CIs) of each covariate. Thus, SCr, Alb, and ALP were not selected as covariates for *V*. In the multivariate analyses with the initial full model, in which PMA, SCr, ALP, and HT were incorporated as covariates for CL, HT was excluded because of its limited impact on OFV. The presence of concomitant medications affecting renal function was not considered because SCr was used as a covariate.

The final PK model was determined as follows (Table 2): *V*(L/kg) = θ*_V_* × exp(η*_V_*) and CL (L/h/kg^0.75^) = θ_CL_ × (PMA/29)^θPMA^ × (SCr/0.64)^θSCr^ × (ALP/958)^θALP^ × exp(η_CL_). The η*i* was assumed to be normally distributed, with a mean of 0 and a variance of ω^2^. The magnitudes of η shrinkage of the final model parameters were 4.80% for CL and 16.0% for *V*, and the ε shrinkage was 5.91%.

**TABLE 2:** Summary of key univariate population PK model building process

Model description^*a*^	Population model^*b*^	OFV^*c*^	ΔOFV^*d*^
CL (base model)	CL = θ_CL_ × (WT)^0.75^	819.6	
SCr	CL = θ_CL_ × (SCr/0.64)^θCL-SCr^ × (WT)^0.75^	678.8	−140.8
PMA	CL = θ_CL_ × (PMA/29)^θCL-PMA^ × (WT)^0.75^	509.4	−310.2
PNA	CL = θ_CL_ × (PNA/6)^θCL-PNA^ × (WT)^0.75^	585.3	−234.3
Alb	CL = θ_CL_ × (Alb/2.7)^θCL-Alb^ × (WT)^0.75^	817.3	−2.3
HT	CL = θ_CL_ × (HT/31)^θCL-HT^ × (WT)^0.75^	656.7	−162.9
ALP	CL = θ_CL_ × (ALP/958)^θCL-ALP^ × (WT)^0.75^	716.9	−102.7

*V* (base model)	*V* = θ*_V_* × (WT)^1.0^	819.6	
SCr	*V* = θ*_V_* × (SCr/0.64)^θ^*^V^*^-SCr^ × (WT)^1.0^	797.8	−21.8
PMA	*V* = θ*_V_* × (PMA/29)^θ^*^V^*^-PMA^ × (WT)^1.0^	816.2	−3.4
PNA	*V* = θ*_V_* × (PNA/6)^θ^*^V^*^-PNA^ × (WT)^1.0^	812.0	−7.6
Alb	*V* = θ*_V_* × (Alb/2.7)^θ^*^V^*^-Alb^ × (WT)^1.0^	806.8	−12.8
HT	*V* = θ*_V_* × (HT/31)^θ^*^V^*^-HT^ × (WT)^1.0^	819.6	0
ALP	*V* = θ*_V_* × (ALP/958)^θ^*^V^*^-ALP^ × (WT)^1.0^	806.1	−13.5

a CL, clearance; *V*, volume of distribution; PNA, postnatal age; PMA, postmenstrual age; HT, current height; SCr, serum creatinine; Alb, albumin; HT, current height; ALP, alkaline phosphatase.

b WT, current body weight.

c OFV, objective function value.

d ΔOFV, change in OFV from the base mode.

### Model validation.

**FIG 2:**
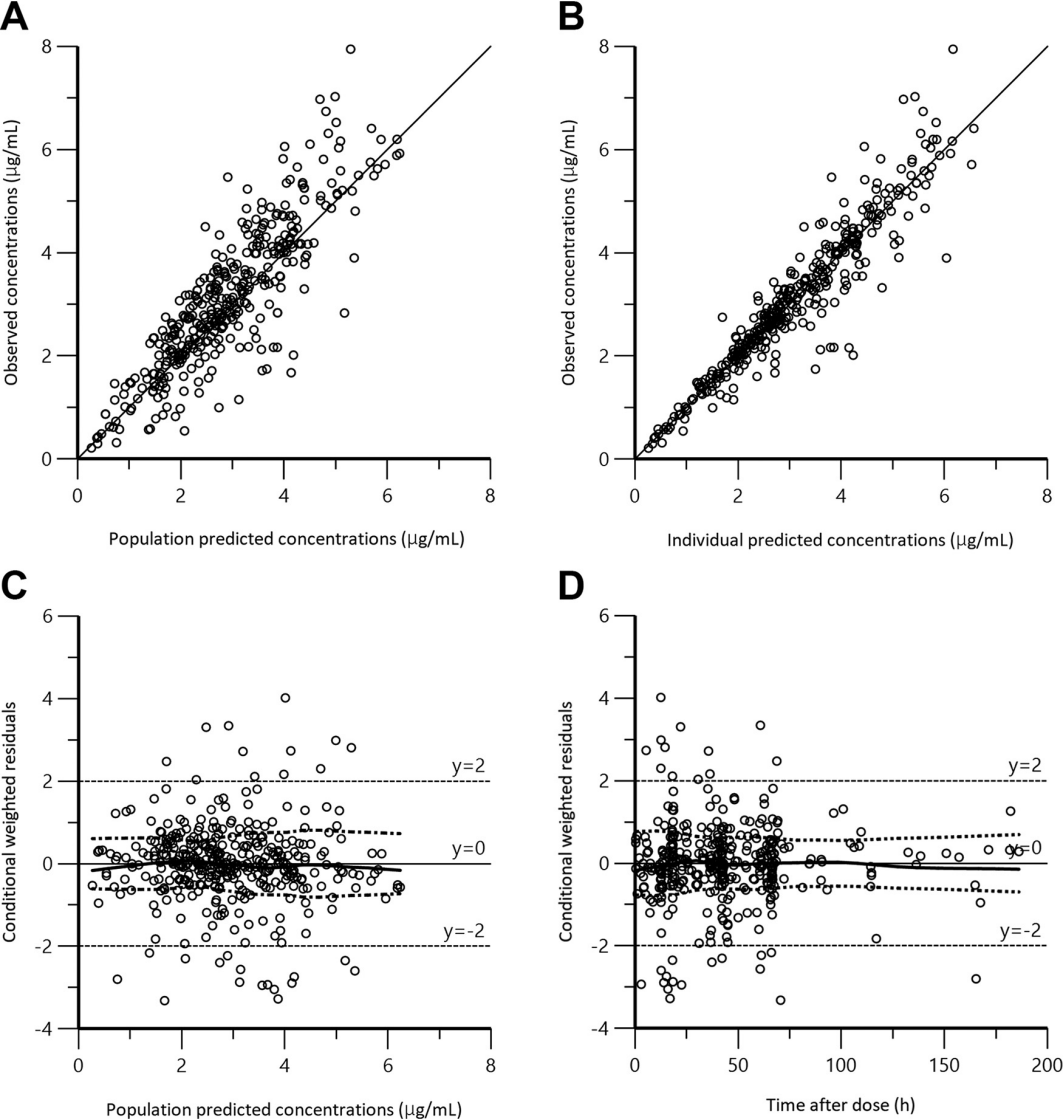
Goodness-of-fit plots for the final population pharmacokinetic model. (A) Plot of observed FLCZ concentrations (μg/mL) versus PRED (μg/mL). (B) Plot of observed FLCZ concentrations (μg/mL) versus IPRED (μg/mL). (C) Plot of CWRES versus PRED. (D) Plot of CWRES versus time after the last dose. (A, B) The solid line is the line of unity (*y* = *x*). (C, D) The top dotted curve is a locally weighted scatterplot smoothing fitted to the absolute values of the residuals, and the bottom dotted curve depicts the top dotted curve above the *x* axis. The solid middle line is the locally weighted scatterplot smoothing fitted to the raw residuals.

**FIG 3:**
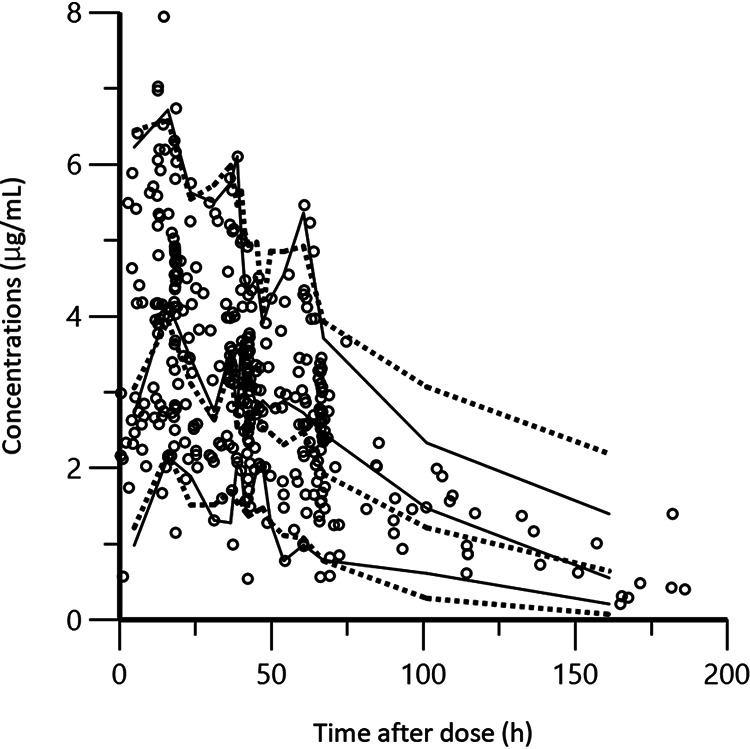
Visual predictive check of observed and simulated concentrations. Visual predictive checks (*n* = 1,000) of the observed FLCZ concentrations (μg/mL) along with the 5th, 50th, and 95th percentiles overlaid on the median and 90% prediction intervals of simulated concentrations generated from the final model. The solid lines are the 5th, 50th, and 95th percentiles of the observed concentrations. The dotted lines are the median and 90% prediction intervals of simulated concentrations.

Table 3 illustrates the statistical distributions of the parameter estimates obtained via bootstrap analyses. The final model converged in 1,000-bootstrap samples, with a convergence rate of 100%. The median values of the parameters estimated via bootstrap analyses were in agreement with the estimated population parameters, and the 95% CIs were narrow, indicating satisfactory precision. The basic goodness-of-fit plots of the final model revealed that the scatterplots of the observed concentrations versus the population predicted concentration (PRED) and the observed concentrations versus the individual predicted concentration (IPRED) each had a uniform distribution around the line of identity (Fig. 2A and B). In addition, the values of the conditional weighted residuals (CWRES) had a symmetrical distribution around zero across the entire PRED and time-after-dose ranges (Fig. 2C and D). The predictive performance of the model observed via visual predictive check is shown in Fig. 3. The 5th, 50th, and 95th percentiles of observed concentrations were close to the respective percentiles of the simulated concentrations.

**TABLE 3:** Population pharmacokinetic estimates for the final model and bootstrap analyses

Parameter	Base model		Final model		Bootstrap value of final model	
	Estimate	(% RSE)^*e*^	Estimate	(% RSE)	Median	95% CI^*f*^
CL (L/h/kg^0.75^)^*a*^						
θ_CL_	0.011	(10.59)	0.011	(10.59)	0.011	0.0091 to 0.015
θ_PMA_			1.52		1.53	1.28 to 3.31
θ_SCr_			−0.17		−0.17	−0.31 to −0.08
θ_ALP_			0.10		0.10	0.018 to 0.24
*V* (L/kg)^*b*^						
θ*_V_*	0.93	(5.40)	0.95	(5.40)	0.95	0.86 to 1.04
*k_c_* (per h)^*c*^						
θ*_kc_*	0.43		0.43	(5.40)	0.43	0.43 to 0.43

Interindividual variance (CV%)^*d*^						
CL	44.3	(34.8)	28.1	(34.8)	26.7	13.4 to 35.2
*V*	14.1	(49.2)	15.8	(49.2)	15.1	2.8 to 21.1

Residual variance						
Proportional (CV%)	21.9	(12.8)	14.0	(12.8)	14.0	10.5 to 17.5
Additive (μg/mL)	0.11	(70.7)	0.068	(70.7)	0.068	0.0011 to 0.16

a CL (L/h/kg^0.75^) = θ_CL_ × (PMA/29)^θPMA^ × (SCr/0.64)^θSCr^ × (ALP/958)^θALP^.

b *V* (L/kg) = θ*_V_*.

c *k_c_* (per h) = θ*_kc_*.

d CV, coefficient of variation.

e RSE, residual standard error.

f CI, confidence interval.

### Dosing simulation.

Monte Carlo simulations were performed using the final model to explore the dosing regimens corresponding to those of the ELBWIs. The results of the Monte Carlo simulation using the standard dosing regimen are shown in Table 4. When a dose of 3 mg/kg of body weight every 72 h was adopted as the dosing regimen, the median trough FLCZ concentrations at steady state (5th to 95th percentile) were 3.5 (2.1 to 5.6), 2.7 (1.6 to 4.1), and 2.1 (1.2 to 3.2) μg/mL among infants aged ≤28, 29 to 36, and ≥37 weeks PMA, respectively. The maximum FLCZ concentration at steady state was <25 μg/mL in all three PMA groups. The values for probability of target attainment (PTA), which were determined by counting the number of patients who achieved a trough concentration of >2 μg/mL throughout the dosing interval, were 95.8%, 88.9%, and 43.3%, respectively, in the three PMA groups specified above. The PTA was dependent on the PMA and the SCr levels. The PTA was >90% for all infants in the <28-week PMA group, regardless of the SCr levels. In the 29- to 36-week PMA group, the PTA was >90% only in infants with ≥0.5 mg/dL SCr. In the ≥37-week PMA group, the PTA was >90% only in infants with ≥1.6 mg/dL SCr. At a dose of 3 mg/kg every 48 or 24 h applied to infants with ≥29-week PMA, both the 29- to 36-week PMA and ≥37-week PMA groups achieved PTA of 90%.

**TABLE 4:** Monte Carlo simulations of the standard dosing regimen for fosfluconazole

Dose of F-FLCZ as FLCZ equivalent, parameter^*a*^	Value for infants with PMA (wks) of^*b*^:		
	≤28	29–36	≥37
3 mg/kg q72h			
Median *C*_ss_ (5th–95th) (μg/mL)			
Trough	3.5 (2.2–4.9)	2.7 (1.7–4.0)	2.1 (1.3–3.0)
Maximum	6.6 (5.3–8.0)	5.9 (4.9–7.1)	5.2 (4.4–6.1)
PTA (%)			
Overall	95.8	88.9	43.3
With SCr (mg/dL) of:			
<0.5	99.2	50.0	5.6
0.5–1.0	100.0	94.4	38.9
1.1–1.5	100.0	100.0	77.8
1.6–2.0	100.0	100.0	94.4
>2.0	100.0	100.0	100.0

3 mg/kg q48h			
Median *C*_ss_ (5th–95th) (μg/mL)			
Trough	5.8 (3.9–8.0)	4.7 (3.0–5.9)	3.7 (2.5–5.1)
Maximum	9.0 (7.0–11.1)	7.9 (6.3–9.7)	6.9 (5.6–8.2)
PTA (%)	100.0	100.0	100.0

3 mg/kg q24h			
Median *C*_ss_ (5th–95th) (μg/mL)			
Trough	13.7 (9.1–16.9)	10.8 (7.6–14.6)	8.8 (6.2–11.6)
Maximum	16.2 (12.2–20.5)	13.9 (10.7–17.7)	11.9 (9.3–14.7)
PTA (%)	100.0	100.0	100.0

a F-FLCZ, fosfluconazole; FLCZ, fluconazole; q72H, every 72 h; *C*_ss_, concentration of fluconazole at steady state; 5th–95th, 5th to 95th percentile; PTA, probability of target attainment; SCr, serum creatinine.

b PMA, postmenstrual age.

## DISCUSSION

Several off-label drugs are used for infants, including F-FLCZ. PK studies are essential for the development of optimal dosing regimens in this population. This study is the first to assess the FLCZ PK in ELBWIs treated with F-FLCZ by developing a population PK (PPK) model. In terms of the results of the PK analysis, the mean CL and *V* in our results were 0.011 L/h/kg^0.75^ and 0.95 L/kg, respectively, comparable with those reported in previous studies on infants with a mean PMA of 28 weeks (CL, 0.0127 L/h/kg^0.75^; *V*, 1.0 L/kg) (14).

The model building process of FLCZ PK confirmed that PMA, SCr, and ALP could be the covariates of CL. Among these covariates, PMA had the greatest effect on FLCZ CL, and the CL of infants with a PMA of 28 weeks was reduced by 50% compared with that of infants with a PMA of 42 weeks. The CL of drugs is affected by the maturation of organ function (16), and PMA has been reported as a good predictor for the CL of primarily renally eliminated drugs in patients with normal renal function (17). In contrast, changes in the SCr levels showed a smaller effect on CL than did PMA. The covariate coefficient of SCr was −0.17, suggesting that the CL was decreased by 10%, with SCr levels increased by 1.0 mg/dL. Although SCr was also included as a covariate for CL of renally eliminated drugs in previous PPK studies on neonates and infants (14, 15), SCr in infants during the first few weeks of life might not be a reliable indicator of renal function (18, 19), and SCr as the covariate for CL accounted only for deviation from the normal renal functions in the WT- and PMA-based models (17). A figure presenting results suggesting a variation in FLCZ CL with SCr has been included in the supplemental material (Fig. S1). In this study, although the actual renal function of infants in the first few weeks was unknown, the FLCZ CL of infants with SCr of ≥1.0 mg/dL was significantly lower than that of infants with SCr of <1.0 mg/dL (the mean CL values were 0.008 mL/h/kg^0.75^ and 0.012 mL/h/kg^0.75^, respectively). This finding explains why SCr was included as a covariate for CL.

Through the PPK modeling process, 76.2% (115/151) of SCr values were collected within 28 days of life. The accuracy of the predicted FLCZ concentrations did not differ by PNA: the root mean square error values of the predicted and observed FLCZ levels were 0.56 for a PNA of ≤7 days, 0.50 for PNAs of 8 to 14 days, 0.50 for PNAs of 15 to 21 days, 0.47 for PNAs of 22 to 28 days, and 0.59 for PNAs of ≥29 days, suggesting that the created model had good predictability regardless of the number of days after birth (Fig. S2). Even though SCr is not a reliable indicator of renal function, good predictions were obtained at the sampling point immediately after birth. No abnormal SCr in the infant’s mother might explain these results (IQR, 0.47 to 0.51 mg/dL). PPK modeling was conducted using SCr of infants ranging from 0.24 to 2.3 mg/dL and PMA from 22.9 to 37.2 weeks. When deviating from this population, we should remain wary of applying the created model to the FLCZ PK prediction, and further study is warranted to confirm the validity of the use of SCr for FLCZ CL prediction in infants.

The influence of ALP on FLCZ CL after F-FLCZ administration was not clearly understood in the study on adults (20). The covariate coefficient of ALP was 0.1. Even if ALP increases from 100 IU/L to 2,000, which is a reported range of ALP values in preterm infants (21), the CL increases by only 30%. Future studies are warranted to elucidate the effect of ALP on CL.

The target pharmacodynamic (PD) parameter that can prevent fungal infection has been suggested to be a trough FLCZ concentration of >2 μg/mL (14). In a previous study conducted in Japan, Candida albicans and Candida parapsilosis accounted for 70% of Candida infections, with the MICs during 2008 to 2013 being 0.0625 to 0.25 μg/mL and 0.125 to 2.0 μg/mL, respectively (22). In addition, the MICs of C. albicans and C. parapsilosis isolates collected for 3 years from 2018 to 2020 in our hospital were 0.125 to 0.5 μg/mL and 0.5 to 2.0 μg/mL, respectively. As most of the Candida species isolates confirmed through blood culture in our hospital were C. albicans or C. parapsilosis, a trough FLCZ concentration of >2 μg/mL appeared to be appropriate as a target concentration in terms of prophylaxis in infants. The results of the Monte Carlo simulations suggested that the currently recommended F-FLCZ dosage might be insufficient, depending on the PMA and SCr levels. The current regimen (3 mg/kg every 72 h) did not achieve a trough FLCZ concentration of >2 μg/mL in infants with a PMA of ≥37 weeks irrespective of the SCr levels or in infants with a PMA of 29 to 36 weeks that had an SCr level of <0.5 mg/dL. Shortened dosing intervals (i.e., every 48 or 24 h) might be required. In this study, the maximum concentration after F-FLCZ for prophylactic treatment was set at 25 μg/mL, considering the safety of F-FLCZ. The simulated maximum FLCZ concentration was <25 μg/mL in all groups, suggesting that the shortened administration time is acceptable. Based on our results, the dosages of F-FLCZ in Table 5 might be more beneficial to optimize FLCZ exposure among infants.

**TABLE 5:** Recommended dosing regimen for fosfluconazole as fluconazole equivalent

SCr (mg/dL)^*a*^	Regimen (3 mg/kg of body weight at indicated intervals) in infants with PMA (wks) of^*b*^:		
	≤28	29–36	≥37
<0.5	q72h	q48h	q48h
0.5–1.0	q72h	q72h	q48h
1.1–1.5	q72h	q72h	q48h
>1.5	q72h	q72h	q72h

a SCr (serum creatinine) range is 0.24 to 2.3 mg/dL.

b PMA (postmenstrual age) range is 22.9 to 37.2 weeks.

Hepatotoxicity is a safety concern in FLCZ administration (23, 24). In this study, the serum AST of two patients with neonatal asphyxia was >250 IU/L. In both patients, the serum AST level decreased over a period of time and returned to normal during the FLCZ prophylactic administration period. This finding indicates that the temporary elevation of the serum AST level might not have been due to FLCZ and was possibly attributable to severe neonatal asphyxia. The serum AST levels in the remaining 16 patients were <135 IU/L. The elevations of the liver enzyme levels with FLCZ in children (age, 0 to 17 years) were reported to be <5%, and these elevations were transient (25). The risk of hepatotoxicity due to the prophylactic administration of F-FLCZ appears to be similar to that of FLCZ.

In this study, scavenged samples were used for PK analysis. Dosing and sampling times were recorded precisely. Samples were only included once they had validated sampling information. As the circulating blood volume was limited and the feasibility of blood collection was low, the use of leftover blood samples obtained during routine clinical care was particularly effective in ELBWIs.

The current study has several limitations. First, it was a single-center prospective study, so the generalizability of the results is limited. Second, the relationship between the dosage of F-FLCZ and its efficacy and safety (the presence of infection and the occurrence of adverse effects) could not be sufficiently evaluated due to the limited number of cases. Third, because SCr does not accurately reflect the renal function of infants in the first few weeks of life, our model might not accurately assess FLCZ CL when SCr deviates significantly from the range of this study. Fourth, the PD target was set to 2 μg/mL for Monte Carlo simulations. It might be inappropriate for Candida species other than C. albicans and C. parapsilosis because these organisms could be shown to have higher FLCZ MICs of >2 μg/mL. Fifth, pharmacokinetic parameters were estimated from scavenged samples in this study. Further studies on the verification of FLCZ PK analysis using a scavenged sampling strategy are needed. Finally, the interindividual variability of the conversion rate constant (*k_c_*) could not be estimated due to the limited number of samples during the conversion phase from F-FLCZ to FLCZ.

In conclusion, we identified the FLCZ PK after F-FLCZ administration in ELBWIs, and we developed a PK model considering various factors affecting FLCZ exposure. PMA and SCr affected CL. However, the effect of ALP was insignificant. We conclude that dosage adjustment of F-FLCZ based on PMA and SCr levels is recommended. A larger prospective investigational study is warranted to confirm the appropriateness of our suggested dosage, as well as the efficacy and safety of F-FLCZ for prophylactic treatment of infants.

## MATERIALS AND METHODS

### Study design.

This prospective observational study was conducted in the NICU of the National Center for Child Health and Development (NCCHD), a tertiary children’s hospital in Tokyo, Japan. The patients were enrolled between March 2020 and August 2020.The participants comprised ELBWIs, regardless of their gestational age, with central vascular access who had received F-FLCZ as a prophylactic treatment for invasive fungal infection. The administration of F-FLCZ continued until central vascular catheter removal. The patients intravenously received a 3-mg/kg dose of F-FLCZ (Prodif intravenous solution, 100 mg/1.25 mL; Pfizer) as the FLCZ equivalent, according to the internal protocol of the NICU at our center. According to the standard regimen for intravenous FLCZ, the dosing intervals were modified based on the PNA: intravenous F-FLCZ was administered at a dose of 3 mg/kg every 72 h during the first 2 weeks of life, every 48 h within 3 to 4 weeks of life, and every 24 h after 5 weeks of life (26, 27). Incidentally, no loading dose was administered.

### Data collection.

Data were retrieved from the medical records of all participants. They include gestational age, PMA, PNA, birth weight (BW), WT, HT, sex, concomitant drug therapy, SCr (enzymatic assay), SCr of mothers, and the Alb, ALP, AST, and ALT levels.

### PK sample collection.

An opportunistic sampling strategy was employed. Residual blood samples obtained during daily clinical blood tests from the first day of administration until the seventh day after the end of treatment were used for plasma FLCZ measurement. The blood samples were obtained from an arterial line or the heels. The sampling times recorded in electronic medical charts for each routine clinical practice were collected as the PK sampling times. The infusion time of F-FLCZ was not intervened. The collected serum samples were stored at −80°C for a maximum period of 3 months until the analysis of FLCZ concentration.

### Liquid chromatography-mass spectrometry analysis.

The total serum FLCZ concentration was measured using liquid chromatography (LC)-tandem mass spectrometry (MS/MS) (TSQ Vantage LC-MS with Dionex UltiMate 3000 RSLC system; Thermo Fisher Scientific K.K., Tokyo, Japan) in the hospital’s internal laboratory using a validated method (28). FLCZ (>99.0%) and its internal standard (IS; FLCZ-d4 stable isotope [>99.0%]) were purchased from Toronto Research Chemical (North York, ON, Canada). The developed assay was confirmed via cross-laboratory validation at the Toray Research Center (Tokyo, Japan).

### PK analysis.

Data regarding dosage, sampling time, and demographic characteristics were merged with the bioanalytical information to create the PK data set. The analysis was performed using Phoenix NLME 8.2 software (Certara USA, Inc., Princeton, NJ, USA). The models were evaluated based on a three-step strategy: basic population model selection, covariate selection, and validation (29). The structural model was evaluated as a one- or two-compartment model with first-order conversion. An exponential error model was assumed for interindividual variation, and additive, proportional, and combined error models were considered for residual variation. Interindividual random effects (ω2) were evaluated according to CL and *V*. The goodness-of-fit for a model was assessed based on significant decreases in the −2-log likelihood of OFV, PRED, and IPRED versus observed concentrations and CWRES versus observed concentrations and time (30) and on changes in the standard errors of parameter estimates (precision). Shrinkage was calculated for all model parameters. A shrinkage value of <20% was considered acceptable (31). Demographic and laboratory characteristics, including PMA, PNA, BW, and HT and SCr, Alb, and ALP levels, were used as continuous covariates, and the correlation between covariates was evaluated. Missing covariate values were imputed using the closest value available for participants, and either the carryforward approach or the backfill approach was adopted, depending on which date was the closest. Continuous covariates were utilized in the allometric model using the following equation: *P_i_* = *P*_pop_ × (Cov*_i_*/Cov_median_)^PWR^, where *P_i_* represents the individual parameter estimate of the *i*^th^ patient, *P*_pop_ indicates the population parameter estimates, Cov is the covariate, and PWR (power) is the exponent. CL was scaled using allometric weight (WT^0.75^) and *V* was scaled using weight (WT^1.0^) as a default structure model (16, 32, 33). Each covariate investigated was retained if it improved the fit as evaluated using biological plausibility, and graphical displays were based on the agreement between the observed and predicted drug concentrations, uniformity of CWRES distribution, improvement in precision in parameter estimates, and reduction of OFV by >3.84 (*P* < 0.05). A forward addition/backward elimination approach for covariate selection was used when two or more covariates were found to be significant for CL rate or *V*. A reduction of 7.88 (*P* = 0.005) was required for covariate retention in the final model.

### Model evaluation.

The bootstrapping approach (*n* = 1,000) and a visual predictive check (*n* = 1,000) using Phoenix NLME 8.2 software were applied for the final model qualifications. The reliability of the final PK parameter estimates and their 95% CIs was assessed using the bootstrap approach. Eta (η) and epsilon (ε) shrinkages were applied to assess the reliability of individual estimations and the power to detect model misspecifications in the goodness-of-fit diagnostics (34).

### Dosing simulations.

Monte Carlo simulations (1,000 replicates) were performed using demographic and laboratory characteristics simulated from the same distribution as in this study, so as to determine the dosing regimens toward maintaining trough FLCZ concentrations of >2 μg/mL. This target concentration was selected based on the typical drug MIC of FLCZ for species isolated from Candida infections (22). F-FLCZ was infused at a dose of 3 mg/kg as the FLCZ equivalent for >0.5 h at three dosing intervals (24, 48, and 72 h). In adults, FLCZ can cause seizures and neurotoxicity at FLCZ concentrations of ≥80 μg/mL (35, 36). Although there have been no reports on the maximum concentration of FLCZ for safety in infants, no serious adverse effects have been reported with a loading dose of 25 mg/kg (23, 37). The mean *V* of FLCZ in infants is 1.0 L/kg based on previous reports (14, 15), and its interindividual variability was within 10%. Theoretically, when a loading dose of 25 mg/kg is administered, the maximum concentration will reach approximately 25 μg/mL (and the maximum concentration shall not greatly exceed this value). The reasoning suggests that the maximum concentration for the safety assessment after F-FLCZ treatment be set to 25 μg/mL. The PTA was determined by counting the number of simulated infants that achieved the trough concentration of >2 μg/mL of FLCZ. Dosing recommendations were determined by identifying the regimens with the smallest total daily dose that achieved the pharmacodynamic target in at least 90% of simulated infants (PTA ≥ 90%).

### Ethical approval.

The study was approved by the Ethics Committee of NCCHD (2019-120) and was conducted in accordance with the Declaration of Helsinki. Informed consent was obtained from persons with parental authority.
