# Supplementary material for: Fluconazole Population Pharmacokinetics after Fosfluconazole Administration and Dosing Optimization in Extremely Low-Birth-Weight Infants
Source: Microbiol Spectr. 2022 Mar 10;10(2):e01952-21. doi: 10.1128/spectrum.01952-21 (PMC9045325; doi:10.1128/spectrum.01952-21)
Supplement: SUPPLEMENTAL FILE 1 — Supplemental material. Download SPECTRUM01952-21_Supp_1_seq6.pdf, PDF file, 0.2 MB [file spectrum01952-21_supp_1_seq6.pdf]

Supplementary Material

Fluconazole Population Pharmacokinetics after Fosfluconazole Administration and Dosing Optimization in Extremely-low-birth-weight Infants

Ayano Tanzawa, Jumpei Saito, Kensuke Shoji, Yuka Kojo, Takanori Funaki, Hidehiko Maruyama, Tetsuya Isayama, Yushi Ito, Hidefumi Nakamura, Akimasa Yamatani.

Supplement Figure 1

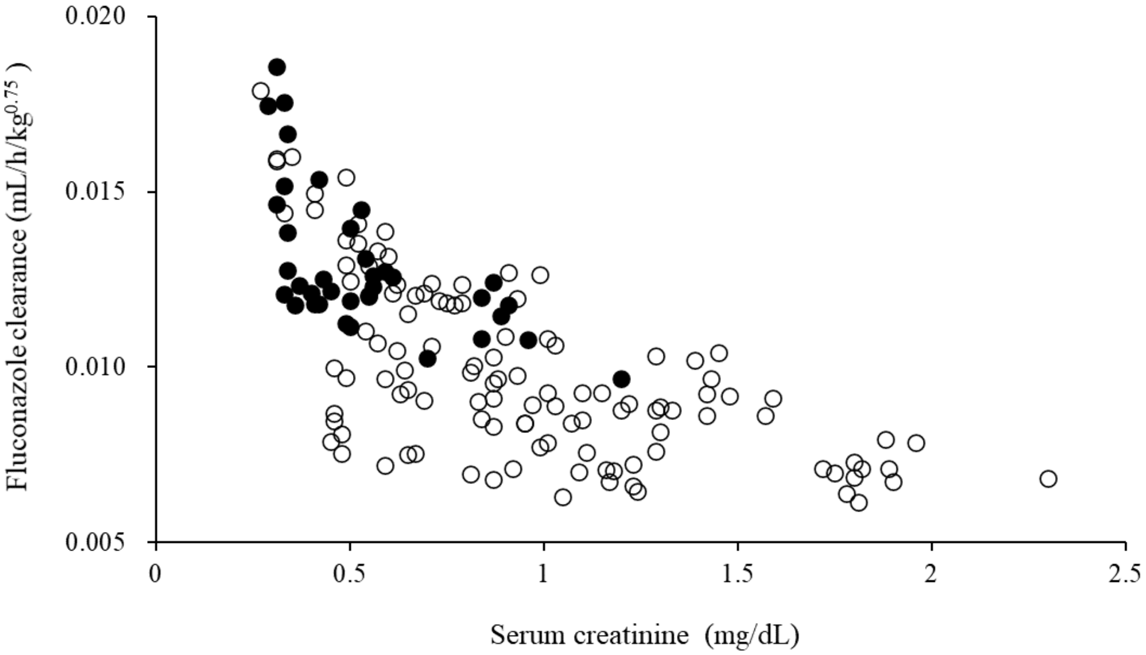

Fig S1 Plots of fluconazole clearance versus serum creatinine. The opened circles indicate infants aged ≤28 days, the closed circles indicate infants aged >28 days.

Supplement Figure 2

(A) Plots of observed FLCZ concentrations (μg/mL) versus PRED (μg/mL) for PNA ≤7 days.

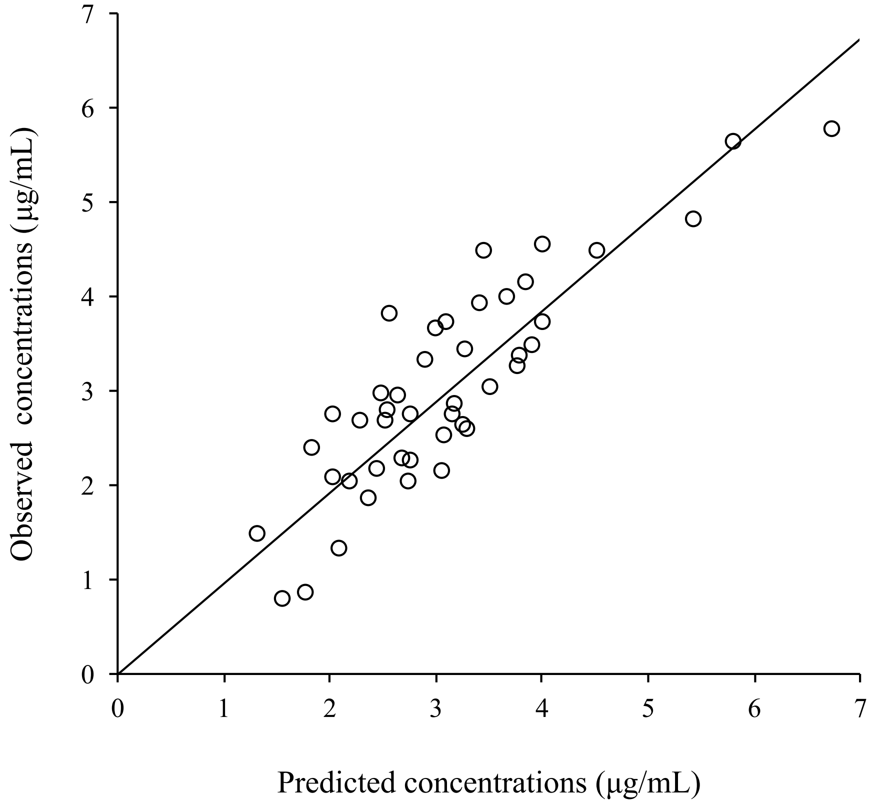

(B) Plots of observed FLCZ concentrations (μg/mL) versus PRED (μg/mL) for PNA from 8 to 14 days.

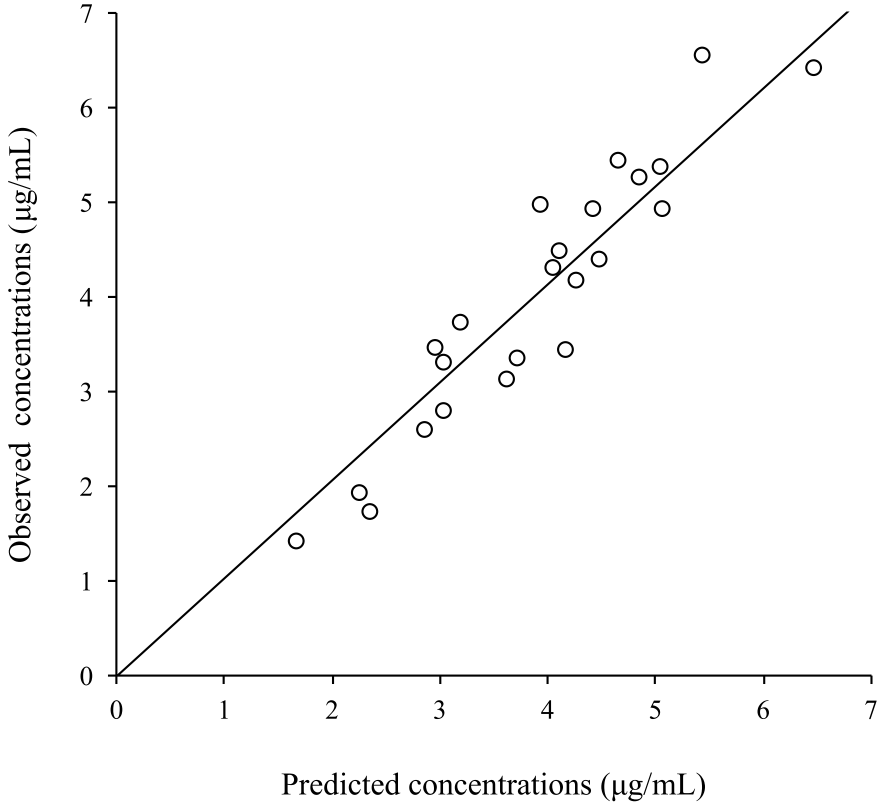

(C) Plots of observed FLCZ concentrations (μg/mL) versus PRED (μg/mL) for PNA from 15 to 21 days.

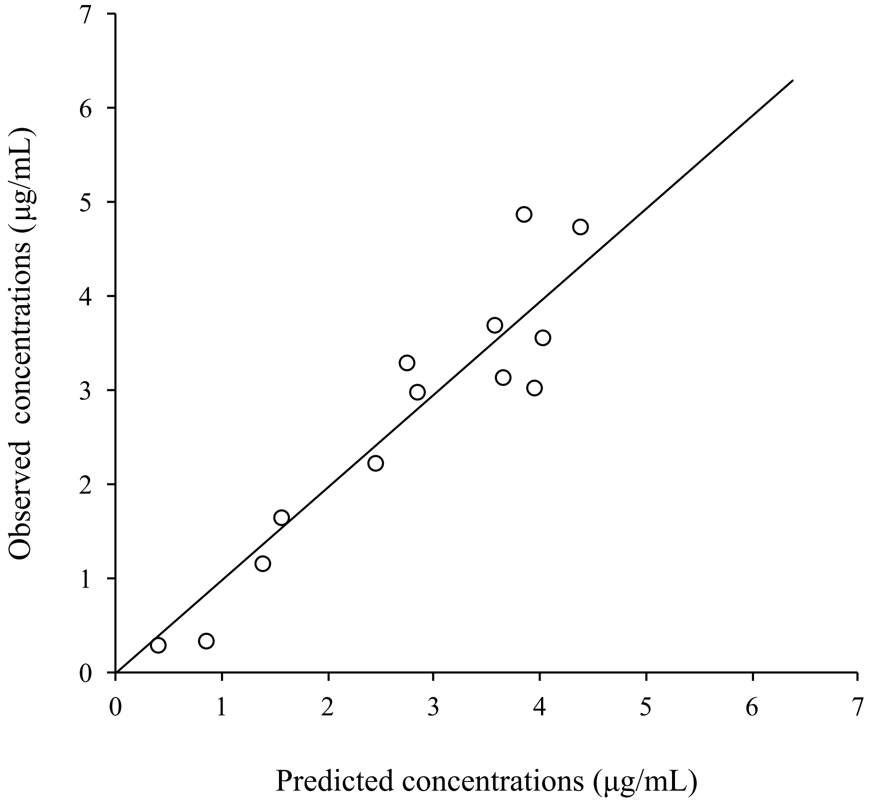

(D) Plots of observed FLCZ concentrations (µg/mL) versus PRED (µg/mL) for PNA from 22 to 28 days.

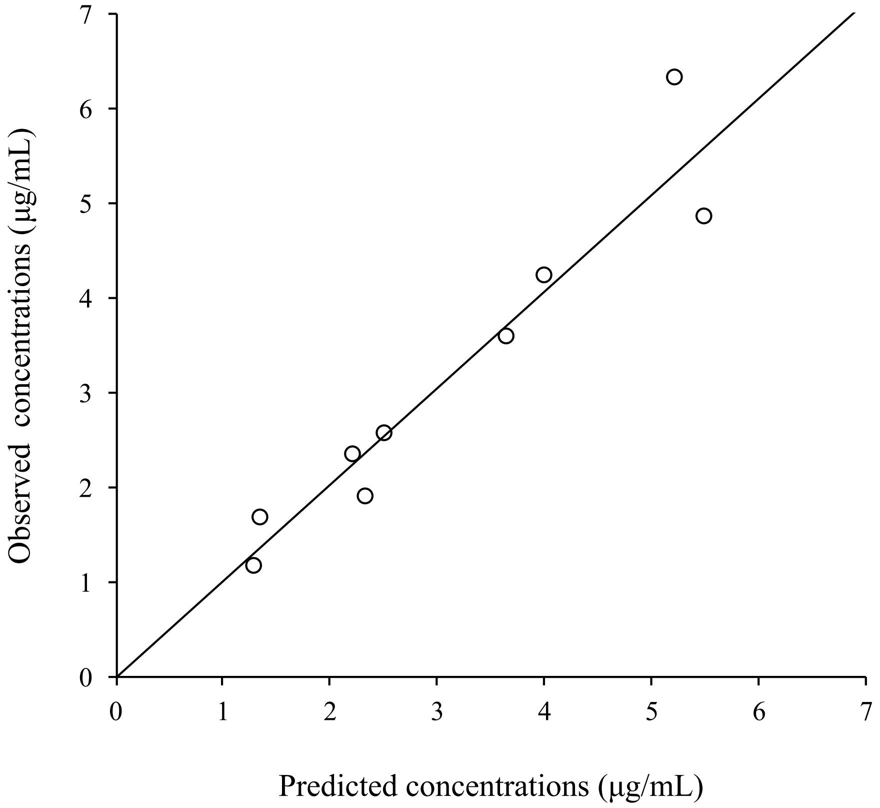

(E) Plots of observed FLCZ concentrations (μg/mL) versus PRED (μg/mL) for PNA ≥29 days.

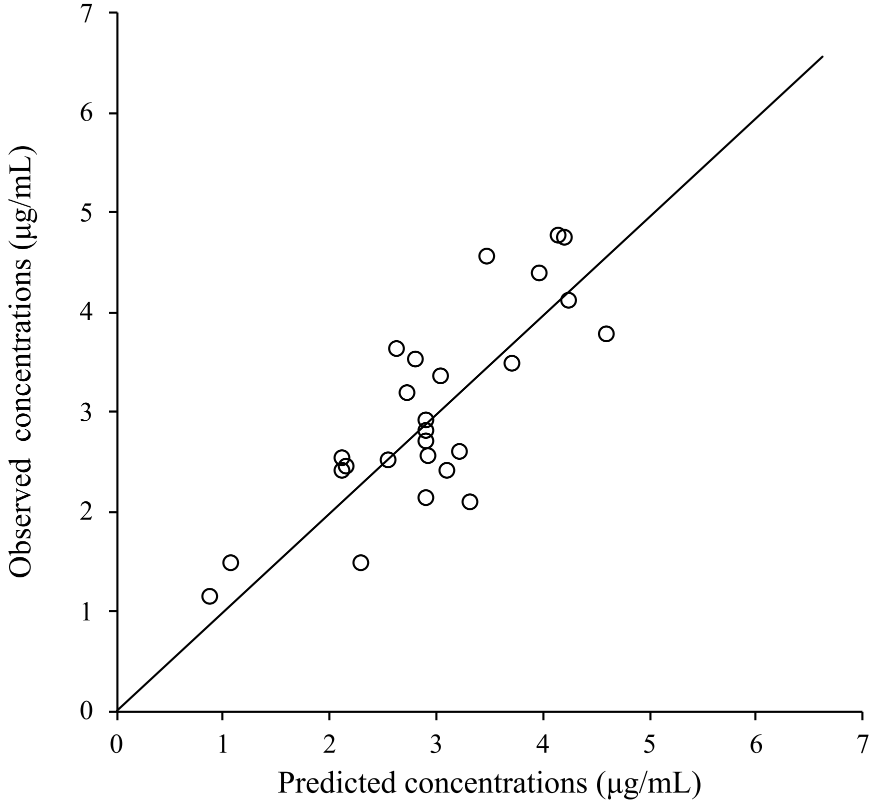

Fig S2 Goodness-of-fit plots for the final model by PNA at the point when SCr values were collected. (A) The plots of observed FLCZ concentrations (μg/mL) versus PRED (μg/mL) for PNA ≤7 days. (B) The plots of observed FLCZ concentrations versus PRED for PNA from 8 to 14 days. (C) The plots of observed FLCZ concentrations versus PRED for PNA from 15 to 21 days. (D) The plots of observed FLCZ concentrations versus PRED for PNA

from 22 to 28 days. (E) The plots of observed FLCZ concentrations versus PRED for PNA ≥29 days. The solid line is the line of unity (y = x).
